# U-shaped association of serum vitamin A concentrations with all-cause mortality in patients with NAFLD: results from the NHANES database prospective cohort study

**DOI:** 10.3389/fnut.2024.1467659

**Published:** 2024-10-30

**Authors:** Hui Li, Jiayuan Ye, Yitian Dong, Weiliang Kong, Guoqing Qian, Yilian Xie

**Affiliations:** ^1^Health Science Center, Ningbo University, Ningbo, Zhejiang, China; ^2^Department of Infectious Diseases, Shangyu People's Hospital of Shaoxing, Shaoxing, Zhejiang, China; ^3^Department of Respiratory and Critical Care Medicine, The First Affiliated Hospital of Ningbo University, Ningbo, Zhejiang, China; ^4^Department of Infectious Diseases, The First Affiliated Hospital of Ningbo University, Ningbo, Zhejiang, China; ^5^Department of Hepatology, The First Affiliated Hospital of Ningbo University, Ningbo, Zhejiang, China

**Keywords:** serum vitamin A, all-cause mortality, NAFLD, NHANES, nonlinear

## Abstract

**Background:**

Previous studies have demonstrated a significant association between serum vitamin A concentration and non-alcoholic fatty liver disease (NAFLD) development. However, the long-term prognostic implications of serum vitamin A in patients with NAFLD remain underexplored. This study aims to investigate whether there exists a correlation between serum vitamin A concentrations and overall mortality among subjects diagnosed with NAFLD.

**Methods:**

To investigate the association between serum vitamin A concentrations and NAFLD outcomes, we conducted prospective cohort studies using data from the 1999–2006 and 2017–2018 National Health and Nutrition Examination Survey (NHANES). We utilized a multivariate Cox regression model to explore the relationship between serum vitamin A levels and all-cause mortality. Survival curves related to serum vitamin A were constructed using the Kaplan–Meier method. Additionally, the restricted cubic splines (RCS) method was applied to examine potential nonlinear relationships between serum vitamin A concentrations and all-cause mortality of NAFLD.

**Results:**

Over a median follow-up period of 10.3 years, a total of 1,399 all-cause deaths were recorded. The weighted average concentration of serum vitamin A was 61.48 ± 0.37 μg/dL. After adjusting for potential confounders, a significant U-shaped relationship was identified between serum vitamin A concentrations and the risk of all-cause mortality in NAFLD patients. This relationship was particularly pronounced in men and elderly individuals aged 60 to 85.

**Conclusion:**

Our study reveals a significant non-linear relationship between serum vitamin A concentrations and the risk of all-cause mortality in patients with NAFLD. These findings underscore the importance of monitoring and maintaining optimal serum vitamin A levels to potentially improve survival outcomes in NAFLD patients.

## 1 Introduction

Non-alcoholic fatty liver disease (NAFLD) affects approximately 30% of the global population and represents a significant global public health concern due to its increasing prevalence (1, 2). It is defined by fat accumulation in hepatocytes without secondary hepatic steatosis causes, such as excessive alcohol consumption, viral hepatitis, or genetic disorders (3). NAFLD encompasses a spectrum of hepatic damage, ranging from simple steatosis to more severe conditions such as non-alcoholic steatohepatitis (NASH), with or without fibrosis, cirrhosis, and hepatocellular carcinoma (4). Despite its global prevalence, the precise mechanisms underlying the onset and progression of NAFLD remain poorly understood. The multiple parallel hit hypothesis states that NAFLD develops through complex interactions involving insulin resistance, adipokine secretion, oxidative stress, lipid peroxidation, mitochondrial damage, endoplasmic reticulum stress, intestinal microbiota, innate immunity, genetics, and epigenetic mechanisms (4). Oxidative stress and inflammation are believed to play critical roles in the transition from steatosis to NASH (5–7).

Patatin-like Phospholipase Domain Containing 3 (PNPLA3) is a multifunctional enzyme that acts as a triglyceride hydrolase, retinyl esterase, and acetyl-CoA-independent transacylase and promotes the release of retinol from lipid droplets (8–10). Pirazzi et al. reported that PNPLA3 can specifically hydrolyze retinyl palmitate in human hepatic stellate cells (HSCs), with this enzymatic activity significantly reduced in the PNPLA3-I148M variant (11). Other studies indicate that the PNPLA3-I148M variant may lead to lower serum retinol levels in patients with NAFLD, accompanied by hepatic accumulation of retinyl esters and triglycerides (4). Recent genetic studies have demonstrated that the PNPLA3-I148M variant is an independent risk factor for the development and severity of liver fibrosis, regulating the activity of HSCs and leading to a pro-inflammatory and pro-fibrotic phenotype (12). Vitamin A, a vital fat-soluble vitamin essential for human physiology, plays a crucial role in several physiological processes such as vision, cell proliferation, and differentiation, immune regulation, embryogenesis, glucose, and lipid metabolism. Approximately 60–95% of the body’s vitamin A is stored in the form of retinyl esters in HSCs (9, 10). Previous studies have suggested that vitamin A and its metabolites may have therapeutic potential for liver diseases (9, 13). Therefore, we hypothesized that there might be a connection between serum vitamin A levels and NAFLD. Lotfi et al. found that higher vitamin A intake was associated with a lower risk of developing NAFLD (14). Mazidi et al. observed that a higher quartile of serum retinol was associated with a reduced risk of NAFLD (15). Furthermore, several studies have indicated a positive correlation between serum vitamin A levels and the severity of NAFLD (16, 17). However, the relationship between serum vitamin A levels and the long-term prognosis of patients with NAFLD remains insufficiently explored. Based on these findings, we investigated the relationship between serum vitamin A concentrations and all-cause mortality in a nationally representative sample of American NAFLD patients.

## 2 Materials and methods

### 2.1 Study design and subjects

The data utilized in this study were obtained publicly from the National Health and Nutrition Examination Survey (NHANES) database. NHANES is a nationwide survey and examination program conducted by the National Center for Health Statistics (NCHS) under the Centers for Disease Control and Prevention (CDC) in the United States since 1999. All data were collected through household interviews, mobile examinations, and laboratory tests. All participants provided written informed consent. NHANES interviews gather data on demographic characteristics, dietary intake, physical examinations, and laboratory tests to assess disease prevalence, risk factors, and nutritional status among the non-institutionalized civilian population of the United States. For more information on NHANES, please refer to the relevant website.^1^

**Figure 1:**
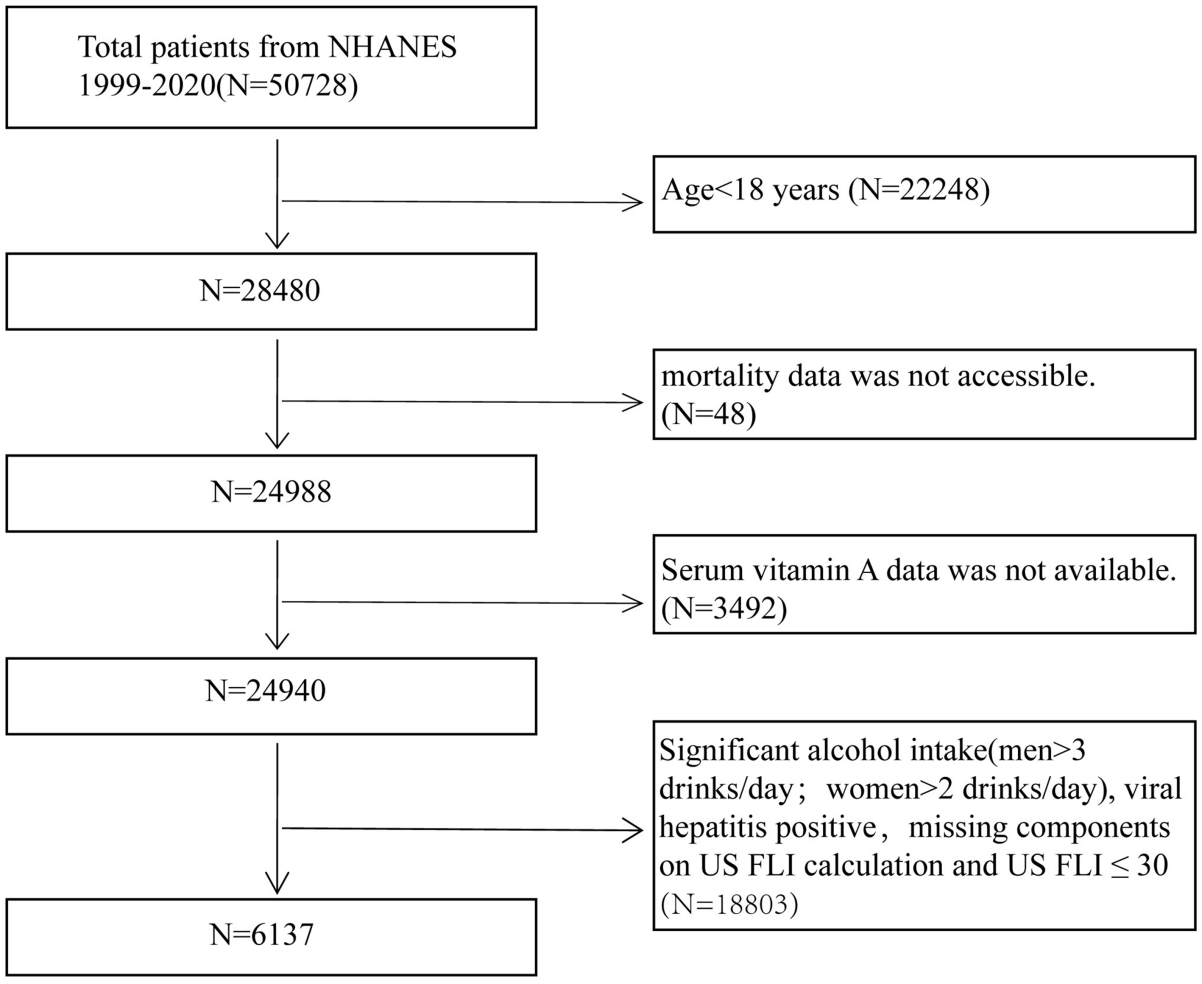
Flow-chart of the study samples.

Data for this study were obtained from the NHANES conducted during 1999–2006 and 2017–2018. Due to the absence of abdominal ultrasound data in the NHANES database, the United States Fatty Liver Index (US FLI) was employed to diagnose NAFLD (18). To ensure the reliability of the study, participants were excluded based on the following criteria: (1) individuals under 18 years of age (*N* = 22,248); (2) those with missing serum vitamin A data (*N* = 3,492); (3) individuals with missing mortality rate data (*N* = 48), and (4) individuals meeting criteria such as excessive alcohol consumption (men >3 drinks/day, women >2 drinks/day), positive hepatitis B or C status, missing US FLI components, or US FLI ≤30 (*N* = 18,803) (18, 19). After applying these exclusion criteria, the final study population comprised 6,137 NAFLD participants. Figure 1 outlines the detailed flowchart illustrating the participant selection process.

### 2.2 Serum vitamin A

Serum samples in this study were collected, processed, and stored according to standardized protocols. Comprehensive details of all assay procedures can be accessed on the official NHANES website. Serum vitamin A concentrations were quantified using high-performance liquid chromatography and photodiode array detection. To explore the association between various serum vitamin A concentrations and all-cause mortality rates among NAFLD patients, the concentrations were divided into four groups by the quartile values: Q1 [0.7, 46.1], Q2 (46.1, 56.8], Q3 (56.8, 69.2], and Q4 (69.2, 185] μg/dL.

### 2.3 Non-alcoholic fatty liver disease

Liver biopsy is recognized as the gold standard for diagnosing NAFLD; however, its use is restricted due to its invasiveness and high cost. Consequently, the improved US FLI and the Fibrosis4 (FIB4) scores were employed to assess NHANES in this study. The US FLI has demonstrated predictive capabilities for hepatic steatosis. US FLI >30 is used to define NAFLD (18). The Fibrosis-4 score (FIB4 score) is employed to evaluate the risk of advanced fibrosis, with a threshold set at 2.67 (20).

The formulas are as follows:

US FLI = (e^−0.8073 × non-Hispanic black + 0.3458 × Mexican American + 0.0093 × age + 0.6151 × ln (GGT) + 0.0249 × waist circumference + 1.1792 × ln (insulin) + 0.8242 × ln (glucose) − 14.7812)^/(1 + e^−0.8073 × non-Hispanic black + 0.3458 × Mexican American + 0.0093 × age + 0.6151 × ln (GGT) + 0.0249 × waist circumference + 1.1792 × ln (insulin) + 0.8242 × ln (glucose) − 14.7812^) × 100 (18, 21).

FIB4 score = Age (year) × AST (IU/L)/(platelet count (109/L) × square-root of ALT (IU/L)) (22).

### 2.4 The mortality data

The mortality data utilized in this study were linked to the National Death Index (NDI), a comprehensive database maintained by the NCHS that covers all deaths in the United States. Each participant’s follow-up time was from the survey date until their date of death or until December 31, 2019. Detailed mortality data in this study can be accessed through the NHANES Public-Use Linked Mortality Files, available at the following web address: https://www.cdc.gov/nchs/data-linkage/mortality-public.htm.

### 2.5 Covariates

Covariates associated with NAFLD include age, sex, race, glycated hemoglobin A1c (HbA1c), C-reactive protein (CRP), aspartate aminotransferase (AST), alanine aminotransferase (ALT), GGT, uric acid, high-density lipoprotein (HDL), low-density lipoprotein (LDL), alkaline phosphatase (ALP), serum cholesterol, serum triglycerides, and energy intake. Race was classified into four groups: non-Hispanic White, non-Hispanic Black, Mexican American, and other races. Body mass index (BMI) was categorized into 3 groups: <25 kg/m^2^ (normal), 25–30 kg/m^2^ (overweight), and ≥ 30 kg/m^2^ (obesity) (23, 24). Smoking behavior was classified as current, former, and never smokers. Drinking behavior was categorized into five groups: (1) never drinkers, (2) mild drinkers (<2 drinks/day for females, <3drinks/day for males), (3) moderate drinkers (≥2 drinks/day for females, ≥3drinks/day for males, or binge drinking ≥2 days/month), (4) heavy drinkers (≥3 drinks/day for females, ≥4 drinks/day for males, or ≥ 4 drinks on a single occasion for females, ≥5 drinks for males), and (5) those with unavailable drinking data (25). Participants’ physical activity levels were categorized into four groups according to the 2018 Physical Activity Guidelines for Americans: low (<500 metabolic equivalent (MET) – minutes per week), moderate (≥500 to <1,000 MET-minutes per week), high (≥1,000 to <1,500 MET-minutes per week), and very high (≥1,500 MET-minutes per week) (26). Diabetes was diagnosed using predefined criteria, including self-report, current use of anti-diabetic medications, HbA1c levels≥6.5%, or fasting blood glucose (FPG) ≥ 126 mg/dL (7 mmol/L) (27). Hypertension was defined as the existence of one of the following conditions: (1) self-reported hypertension, (2) current use of antihypertensive medications, or (3) systolic blood pressure ≥ 140 mmHg or diastolic blood pressure ≥ 90 mmHg (28). Hyperlipidemia was diagnosed if participants met any of the following conditions: (1) triglycerides (TG) ≥150 mg/dL, (2) total cholesterol (TC) ≥ 200 mg/dL, (3) low-density lipoprotein cholesterol (LDL-C) ≥ 130 mg/dL, (4) high-density lipoprotein cholesterol (HDL-C) < 40 mg/dL for males and < 50 mg/dL for females, or (5) receipt of lipid-lowering medication (29).

### 2.6 Statistical analysis

This study’s analyses adhered to the NHANES guidelines and utilized a non-random, stratified sampling design. Continuous variables were presented as weighted means ± standard error (SE) and were examined using weighted linear regression models. Categorical variables were reported as percentages ± SE and were analyzed using weighted Rao-Scott chi-square tests. The multivariate Cox regression analysis was conducted to estimate hazard ratios (HRs) and 95% confidence intervals (CIs) for all-cause mortality in NAFLD patients based on serum vitamin A levels. Three models were developed, each adjusting for different potential confounders: Model 1 without adjustments, Model 2 adjusting for sex, age, and race, and Model 3 further adjusting for BMI, smoking behavior and drinking behavior, physical activity level, energy intake, CRP, diabetes status, and hypertension status. Stratified analyses and interaction tests were performed based on various factors including age groups (18–39, 40–59, 60–85 years), sex (male/female), BMI (<25 kg/m^2^ or ≥ 25 kg/m^2^, <30 kg/m^2^ or ≥ 30 kg/m^2^), diabetes status, hypertension status, and advanced fibrosis status. The association between serum vitamin A levels and survival was illustrated using Kaplan–Meier curves, with comparisons conducted using the log-rank test. Restricted cubic spline (RCS) curves with four nodes (5th, 35th, 65th, and 95th percentiles) were utilized to display the potential non-linear relationships between serum vitamin A levels and all-cause mortality in NAFLD patients. *p*-values ≤ 0.05 were considered statistically significant. Statistical analyses were conducted using the R software (version 4.2.0).

## 3 Results

### 3.1 Basic characteristics of study participants

Table 1 presents the baseline characteristics of the entire study population. The mean age of participants was 50 ± 0.39 years old. The average follow-up period was 10.3 years, culminating in 1399 cases of all-cause mortality. The weighted mean concentration of serum vitamin A was 61.48 ± 0.37 μg/dL. Participants with NAFLD were predominantly obese men, of non-Hispanic white race, with a history of non-smoking or former smoking and mild drinking behavior. Baseline data distribution varied significantly among groups. Compared to those with low serum vitamin A levels, participants with higher levels were more likely to be male, overweight (25–30 kg/m^2^), non-Hispanic White, and have a history of smoking or mild alcohol consumption. Higher serum vitamin A levels were also associated with a greater incidence of hypertension and hyperlipidemia. Additionally, this group showed other metabolic disturbances, including elevated serum ALT, GGT, uric acid, LDL, cholesterol, and triglycerides, while observing an opposing trend for ALP and CRP levels.

**Table 1:** Baseline characteristics of participants according to serum vitamin A concentrations.

Character	Serum vitamin A concentrations (ug/dL)					*p* value
	Total	Q1 [0.7,46.1]	Q2 (46.1,56.8]	Q3 (56.8,69.2]	Q4 (69.2,185]	
Number of participants	6,137 (100)	1,538 (25.06)	1,532 (24.96)	1,534 (25.00)	1,533 (24.98)	
Age (year)	50 (0.39)	44 (0.60)	47 (0.60)	51 (0.63)	55 (0.52)	< 0.0001
BMI (kg/m^2)	31.53 (0.12)	33.88 (0.28)	32.44 (0.21)	31.17 (0.19)	29.66 (0.16)	< 0.0001
HbA1c	5.67 (0.02)	5.75 (0.04)	5.63 (0.03)	5.67 (0.04)	5.65 (0.03)	0.12
ALT (U/L)	27 (0.55)	25 (0.82)	27 (0.54)	28 (0.58)	29 (1.74)	0.03
AST (U/L)	25 (0.23)	23 (0.74)	24 (0.44)	25 (0.45)	25 (0.29)	0.12
GGT (U/L)	33 (0.62)	29 (1.18)	31 (1.18)	32 (1.06)	37 (1.12)	< 0.0001
CRP (mg/L)	0.54 (0.02)	0.86 (0.05)	0.63 (0.05)	0.46 (0.02)	0.39 (0.02)	< 0.0001
Uric acid (mg/dL)	6 (0.03)	5 (0.05)	6 (0.04)	6 (0.04)	6 (0.04)	< 0.0001
HDL (mg/dL)	49 (0.23)	49 (0.46)	48 (0.42)	48 (0.42)	50 (0.41)	< 0.0001
LDL (mg/dL)	122 (0.78)	115 (1.45)	122 (1.53)	125 (1.25)	125 (1.56)	< 0.0001
ALP (U/L)	76 (0.46)	83 (1.09)	76 (0.81)	75 (0.79)	71 (0.83)	< 0.0001
Serum triglyceridel (mg/dL)	167 (2.66)	125 (3.67)	150 (4.33)	169 (2.66)	207 (6.04)	< 0.0001
Serum Cholesterol (mg/dL)	203 (1.02)	189 (1.70)	198 (1.67)	206 (1.52)	215 (1.85)	< 0.0001
Energy intake (kcal/d)	2,180 (18)	1,987 (37)	2,222 (33)	2,202 (39)	2,245 (35)	< 0.0001
Serum VA (μg/dL)	61.48 (0.37)	38.67 (0.20)	51.82 (0.12)	62.82 (0.12)	82.47 (0.43)	< 0.0001
FLI	70.04 (0.39)	70.66 (0.72)	70.67 (0.67)	70.04 (0.74)	69.11 (0.74)	0.39
FIB4 score	1.02 (0.01)	0.90 (0.03)	0.94 (0.02)	1.06 (0.02)	1.12 (0.02)	< 0.0001
Sex						< 0.0001
Male	53.51 (0.02)	30.93 (1.79)	50.43 (1.49)	58.74 (1.56)	65.22 (1.29)	
Female	46.49 (0.02)	69.07 (1.79)	49.57 (1.49)	41.26 (1.56)	34.78 (1.29)	
Race						< 0.0001
Non-Hispanic White	70.83 (0.03)	47.07 (2.36)	67.50 (1.96)	75.81 (1.91)	83.74 (1.44)	
Non-Hispanic Black	11.06 (0.01)	22.40 (1.91)	11.79 (1.19)	8.52 (0.89)	5.75 (0.64)	
Mexican American	7.53 (0.01)	15.08 (1.54)	8.65 (1.01)	6.12 (0.83)	3.21 (0.42)	
Other	10.58 (0.01)	15.44 (1.90)	12.06 (1.35)	9.54 (1.28)	7.30 (1.06)	
BMI category						< 0.0001
<25	8.30 (0.01)	4.23 (0.67)	5.31 (0.83)	8.07 (1.00)	13.55 (1.16)	
≥25, <30	39.81 (0.02)	26.36 (1.56)	36.69 (1.69)	42.65 (1.79)	48.13 (1.82)	
≥30	51.88 (0.02)	69.40 (1.64)	58.00 (1.78)	49.29 (1.83)	38.32 (1.66)	
Physical activity						0.09
Mild	32.08 (0.01)	38.63 (2.22)	39.01 (1.83)	45.93 (2.16)	41.60 (1.86)	
Moderate	14.59 (0.01)	16.99 (1.66)	20.22 (1.62)	17.53 (1.59)	20.41 (1.53)	
High	6.89 (0.01)	9.40 (1.14)	8.22 (1.00)	8.57 (1.06)	9.65 (1.06)	
Very high	23.40 (0.01)	34.97 (2.34)	32.55 (1.92)	27.96 (1.91)	28.35 (1.51)	
Smoking behavior						< 0.0001
Current smoke	16.00 (0.01)	18.02 (1.66)	17.99 (1.41)	15.49 (1.18)	13.57 (1.11)	
Ever smoke	30.38 (0.01)	18.85 (1.94)	27.92 (1.55)	32.13 (1.58)	37.95 (1.71)	
Never smoke	51.86 (0.02)	60.35 (2.35)	51.17 (1.76)	51.23 (1.82)	47.75 (1.50)	
Not recorded	1.76 (0.00)	2.78 (0.38)	2.91 (0.40)	1.16 (0.23)	0.72 (0.17)	
Dinking behavior						< 0.0001
Never drunk	11.90 (0.01)	15.10 (0.97)	13.66 (1.49)	10.24 (1.46)	9.98 (0.85)	
Mild drunk	41.36 (0.02)	30.12 (1.89)	38.41 (1.79)	44.58 (1.97)	47.80 (1.90)	
Moderate drunk	15.70 (0.01)	17.91 (1.53)	17.09 (1.46)	13.52 (1.11)	15.19 (1.29)	
Heavy drunk	1.19 (0.00)	0.31 (0.10)	0.82 (0.34)	1.02 (0.28)	2.19 (0.50)	
Not recorded	29.86 (0.01)	36.56 (1.84)	30.02 (1.73)	30.63 (1.64)	24.85 (1.49)	
Hypertension						< 0.0001
No	51.65 (0.02)	62.90 (1.85)	58.13 (1.72)	49.19 (1.96)	41.55 (1.84)	
Yes	48.35 (0.02)	37.10 (1.85)	41.87 (1.72)	50.81 (1.96)	58.45 (1.84)	
Diabetes						0.25
No	80.57 (0.03)	80.31 (1.22)	84.24 (1.16)	82.62 (1.65)	81.15 (1.29)	
Yes	17.48 (0.01)	19.69 (1.22)	15.76 (1.16)	17.38 (1.65)	18.85 (1.29)	
Hyperlipidemia						< 0.0001
No	16.15 (0.01)	24.88 (1.49)	20.43 (1.48)	15.53 (1.20)	7.74 (1.04)	
Yes	83.85 (0.03)	75.12 (1.49)	79.57 (1.48)	84.47 (1.20)	92.26 (1.04)	
FIB4 score						0.02
≤ 2.67	97.88 (0.03)	97.17 (0.51)	98.75 (0.27)	98.27 (0.32)	98.51 (0.34)	
> 2.67	1.74 (0.00)	2.83 (0.51)	1.25 (0.27)	1.73 (0.32)	1.49 (0.34)	

Data are expressed as weighted proportions ± Standard Error (SE) for categorical variables and as weighted means ± SE for continuous variables. Linear regression and Rao-Scott chi-square test were used to compare groups. BMI, body mass index; HbA1c, glycosylated hemoglobin A1c; ALT, alanine aminotransferase; AST, aspartate aminotransferase; HDL, high-density lipoprotein; LDL, low-density lipoprotein; GGT, gamma-glutamyl transferase; CRP, C-reactive protein; ALP, alkaline phosphatase; FLI, fat liver index; FIB4 score, Fibrosis-4 scores; Serum VA, Serum Vitamin A.

Subsequently, we examined whether there were differences in the severity of liver fibrosis among the four groups, determined by a FIB-4 score. We categorized liver fibrosis into two classifications: non-advanced (≤ 2.67) and advanced (> 2.67). The majority of patients with NAFLD had non-advanced liver fibrosis. The highest proportion of advanced liver fibrosis occurred in patients with low serum vitamin A levels (*p* < 0.05).

### 3.2 Association between serum vitamin A level and all-cause mortality

The study utilized three Cox regression models to explore the independent effect of serum vitamin A levels on all-cause mortality in patients with NAFLD. As illustrated in Table 2, Model 1 revealed a significant association between serum vitamin A levels and an increased risk of all-cause mortality. Specifically, NAFLD patients in the highest serum vitamin A quartile (Q4) exhibited a greater risk of all-cause mortality compared to those in the lowest quartile (Q1). However, after adjusting for relevant variables (Models 2 and 3), serum vitamin A levels were significantly linked to a decreased risk of all-cause mortality among NAFLD patients. Notably, in Model 3, the group with moderate serum vitamin A levels (Q2) had the lowest mortality risk compared to Q1 (HR = 0.633, 95% CI = 0.456–0.880). The groups with higher serum vitamin A levels (Q3 and Q4) also had lower mortality risks than Q1, with Q3 showing HR = 0.727, 95% CI = 0.541–0.976, and Q4 showing HR = 0.663, 95% CI = 0.499–0.880. However, the trend test was insignificant (*p* for trend = 0.077), suggesting a potential nonlinear relationship between serum vitamin A levels and all-cause mortality.

**Table 2:** HRs (95% CIs) for all-cause mortality according to serum vitamin A concentrations among participants.

Serum VA (per SD increase) (μg/dL)	Model 1 OR (95% CI)	*p* value	Model 2 OR (95% CI)	*p* value	Model 3 OR (95% CI)	*p* value
Q1 [0.7,46.1]	Reference		Reference		Reference	
Q2 (46.1,56.8]	0.835 (0.643,1.084)	0.175	0.619 (0.497,0.771)	<0.0001	0.633 (0.456,0.880)	0.006
Q3 (56.8,69.2]	1.230 (0.963,1.570)	0.097	0.668 (0.543,0.821)	<0.001	0.727 (0.541,0.976)	0.034
Q4 (69.2,185]	1.485 (1.181,1.868)	<0.001	0.648 (0.529,0.794)	<0.0001	0.663 (0.499,0.880)	0.004
*p* for trend		<0.0001		0.006		0.077

Model 1: no covariates were adjusted; Model 2: Age, sex, and race were adjusted; Model 3: Adjusted for age, sex, race, BMI, smoking behavior, drinking behavior, physical activity, energy intake, CRP, diabetes, and hypertension status. Abbreviations: HRs, hazard ratios; 95%CI, 95% confidence interval; BMI, body mass index; CRP, C-reactive protein.

### 3.3 Subgroup analysis

**Figure 2:**
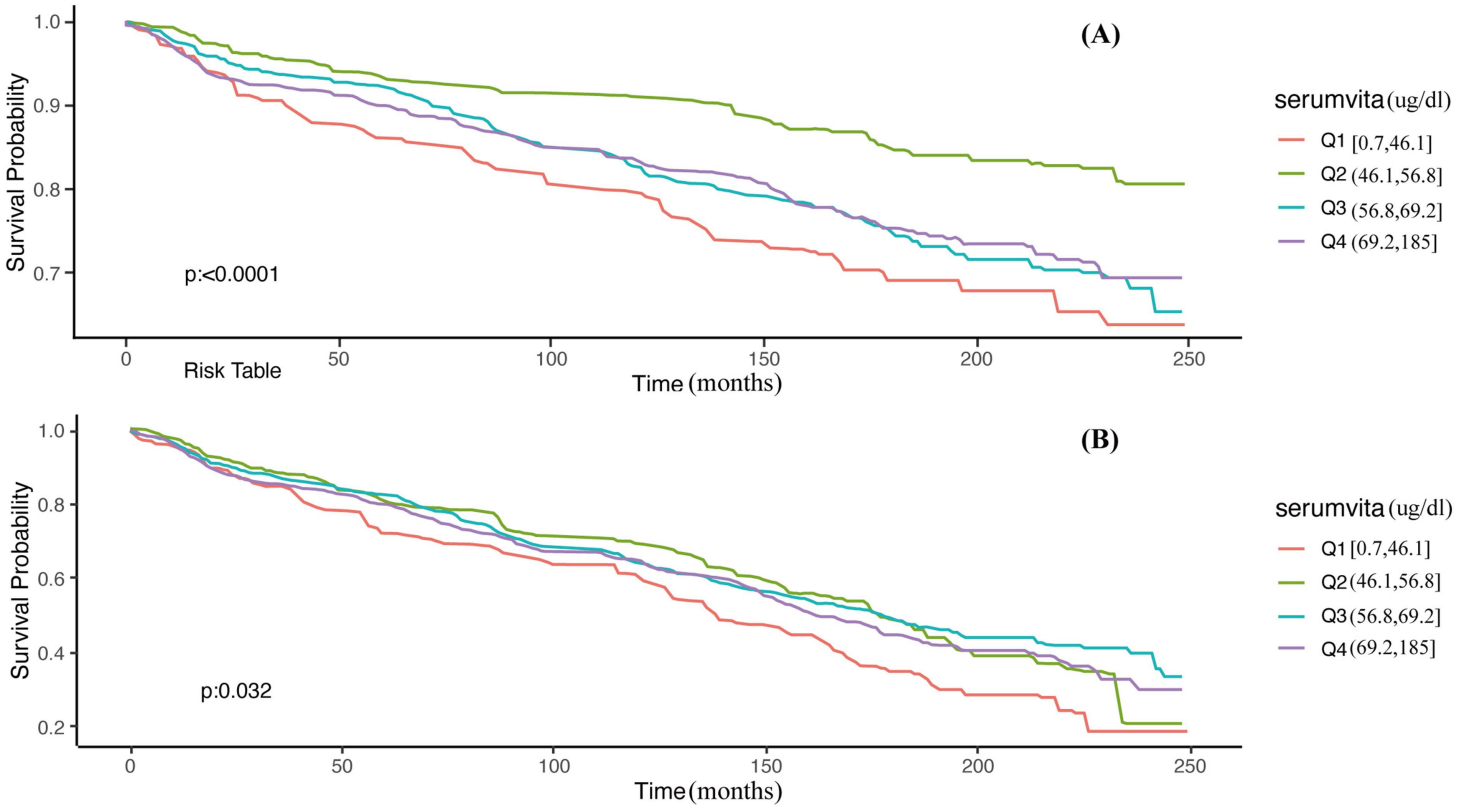
Unadjusted Kaplan–Meier survival curves for all-cause mortality among serum vitamin A. **(A)** Males; **(B)** 60–85 years.

To further elucidate the complex relationship between serum vitamin A levels and all-cause mortality in NAFLD patients, stratified analyses and interaction tests were performed based on sex, age, BMI, diabetes status, hypertension status, and advanced fibrosis status. Details are presented in Table 3. This study demonstrated consistent results when stratified by BMI, diabetes, hypertension, and advanced fibrosis (*p* for interaction >0.05). However, significant interactions were observed when stratified by sex and age (*p* for interaction <0.05), indicating a more pronounced correlation between serum vitamin A levels and all-cause mortality in male and elderly NAFLD patients. Consequently, a thorough examination of the relationship between serum vitamin A and all-cause mortality was conducted across different sex and age categories. Supplementary Tables S1, S2 demonstrate that these associations remain generally consistent among the elderly (60–85 years) and male populations. Additionally, we conducted a Kaplan–Meier analysis on elderly (aged 60–85) and male NAFLD patients, revealing that those in the Q2 group had the lowest risk of all-cause mortality (Log-rank *p* < 0.05), consistent with the Cox regression results (Figure 2).

**Table 3:** Associations between serum vitamin A and all-cause mortality in NAFLD participants, stratified by age, sex, BMI, diabetes status, hypertension status, and advanced fibrosis status.

Subgroup	Q1 [0.7,46.1]	Q2 (46.1,56.8]	Q3 (56.8,69.2]	Q4 (69.2,185]	*p* for trend	*p* for interaction
Sex						0.005
Male	Reference	0.452 (0.280,0.728)	0.765 (0.512,1.144)	0.587 (0.394,0.873)	0.387	
Female	Reference	0.935 (0.615,1.423)	0.599 (0.390,0.922)	0.795 (0.559,1.131)	0.162	
Age						0.018
18–39	Reference	0.500 (0.094, 2.661)	1.128 (0.266, 4.787)	1.219 (0.250, 5.940)	0.481	
40–59	Reference	0.430 (0.203,0.913)	1.044 (0.607,1.794)	0.670 (0.346,1.296)	0.978	
60–85	Reference	0.647 (0.444,0.943)	0.590 (0.417,0.836)	0.617 (0.430,0.884)	0.052	
BMI						0.375
< 25	Reference	1.028 (0.427,2.475)	0.893 (0.389,2.051)	0.626 (0.283,1.384)	0.075	
≥ 25, <30	Reference	0.670 (0.419,1.072)	0.736 (0.504,1.073)	0.685 (0.488,0.963)	0.211	
≥ 30	Reference	0.621 (0.393,0.980)	0.809 (0.534,1.228)	0.750 (0.483,1.166)	0.594	
Diabetes status						0.345
No	Reference	0.610 (0.415,0.896)	0.700 (0.513,0.955)	0.573 (0.430,0.765)	0.002	
Yes	Reference	0.784 (0.411,1.497)	0.886 (0.450,1.743)	1.086 (0.568,2.073)	0.414	
Hypertension status						0.275
No	Reference	0.524 (0.300,0.916)	0.703 (0.419,1.180)	0.498 (0.287,0.865)	0.073	
Yes	Reference	0.705 (0.475,1.047)	0.744 (0.513,1.081)	0.761 (0.540,1.071)	0.428	
Advanced fibrosis						0.719
No (FIB4 score ≤ 2.67)	Reference	0.645 (0.445,0.936)	0.737 (0.517,1.050)	0.696 (0.500,0.969)	0.217	
Yes (FIB4 score>2.67)	Reference	1.110 (0.417,2.953)	1.388 (0.636,3.032)	0.956 (0.342,2.676)	0.855	

Adjusted for age, sex, race, BMI, smoking behavior, drinking behavior, physical activity, energy intake, CRP, diabetes status, and hypertension status, except the variable itself. Abbreviations: 95%CI, 95% confidence interval; BMI, body mass index; CRP, C-reactive protein; FIB4 score, Fibrosis-4 scores.

### 3.4 Dose–response relationship between serum vitamin A levels and all-cause mortality in NAFLD patients

**Figure 3:**
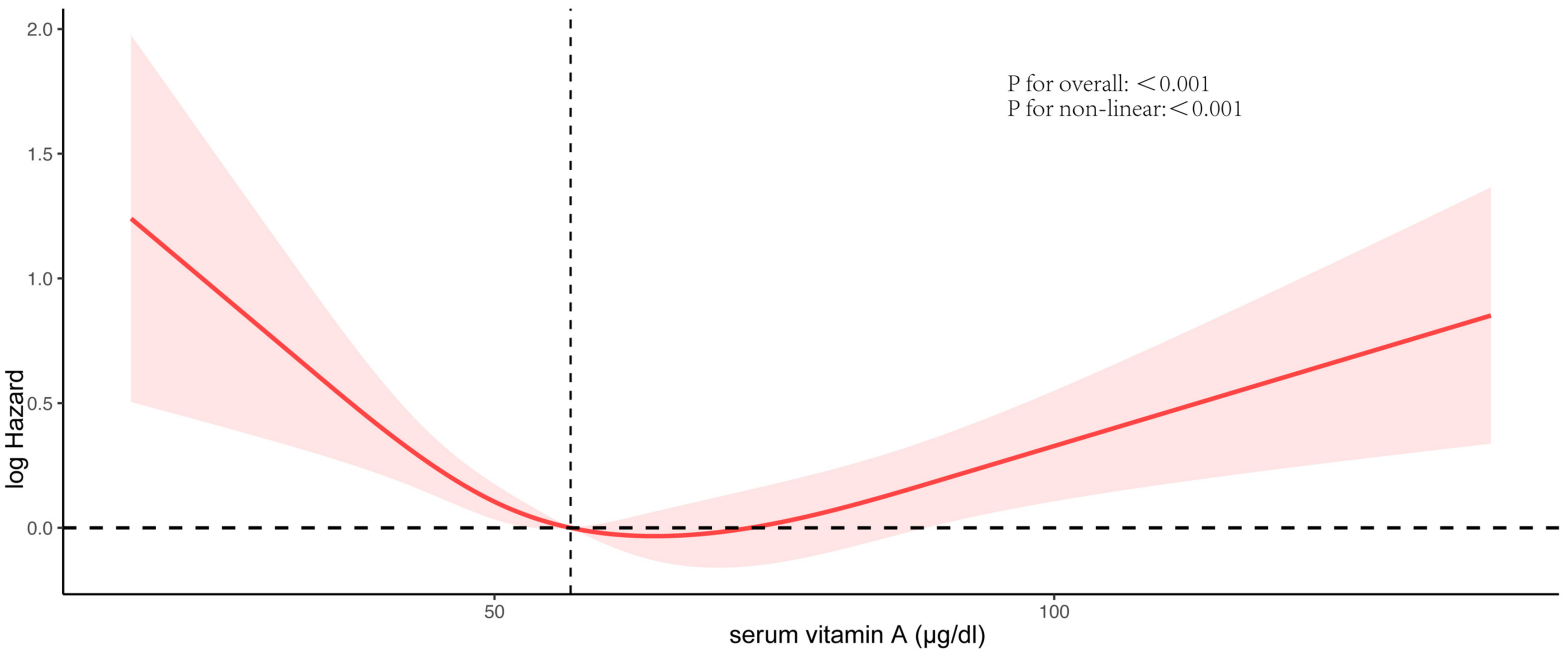
Association between serum vitamin A concentrations and all-cause mortality. Red solid lines and red dotted lines, respectively, represent restricted cubic spline models and 95%CI. Models were adjusted by age, race, BMI, smoking behavior, drinking behavior, physical activity, energy intake, C-reactive protein, diabetes status, and hypertension status.

Figure 3 vividly illustrates the dose–response relationship between serum vitamin A levels and all-cause mortality in NAFLD patients. A notable U-shaped association was identified by applying the RCS model with comprehensive adjustment for all variables (*p* for non-linearity <0.001, *p* for overall <0.001), with a crucial threshold identified at 64.5 μg/dL.

## 4 Discussion

This study employed a prospective cohort design to investigate the relationship between serum vitamin A levels and all-cause mortality in NAFLD patients. The results revealed a U-shaped association between serum vitamin A concentrations and all-cause mortality, indicating that excessively low and high vitamin A levels increase mortality risk. This relationship was particularly pronounced among elderly individuals (aged 60–85) and males. To our knowledge, this is the first study to examine the association between serum vitamin A levels and all-cause mortality in the NAFLD population.

Current research on serum vitamin A levels and mortality rates largely focuses on pediatric populations, with less emphasis on studies involving adults. Abhishek Goyal et al. employed Cox regression analysis to reveal a substantial correlation between serum vitamin A levels and all-cause mortality in the overall population. Their findings indicate a noticeable reduction in mortality risk from Q2 to Q4 in comparison to the initial quintile Q1, while Q5 demonstrates a relative escalation in mortality risk (30). However, they did not conduct an in-depth analysis. An additional investigation discovered a strong correlation between reduced serum retinol levels and heightened occurrences of liver fibrosis and liver-related mortality within a cohort of American adults. For individuals with chronic liver disease (CLD), those in the lowest retinol category exhibited a significantly increased HR for liver-related mortality, reaching 7.76 (95% CI, 1.19–50.5) compared to the highest retinol group. However, no significant difference was observed in all-cause mortality (31). To date, there are no reported clinical studies on the relationship between serum vitamin A levels and all-cause mortality in the NAFLD population. Our study is the first to identify a U-shaped association between serum vitamin A levels and all-cause mortality in individuals with NAFLD.

We conducted stratified analyses to further identify subgroups of NAFLD patients for whom serum vitamin A levels are most strongly associated with all-cause mortality. The results revealed that sex and age are significant influencing factors. Specifically, the association between serum vitamin A levels and the risk of all-cause mortality is more pronounced in elderly individuals (aged 60–85) and males. Previous studies have suggested that NAFLD may be more severe in older populations. For instance, Mazen Noureddin et al. found a significant increase in the prevalence and severity of NAFLD among participants aged 60 or older (32). Frith et al. also observed higher rates of fibrosis and cirrhosis in elderly NAFLD patients (33). Pegah et al.’s study showed a common occurrence of NAFLD in older adults, associated with increased mortality risk in individuals aged 60–74 with NAFLD (29). Furthermore, Sun Q et al. noted that lower serum retinol levels (<50 μg/dL) were linked to increased mortality among participants aged 60 years and older with prediabetes and diabetes, potentially attributed to the increased susceptibility to malnutrition in older age, underscoring the importance of adequate vitamin A intake for nutritional enhancement (34). However, the specific relationship between serum vitamin A levels and all-cause mortality risk in older adults requires further investigation. Additionally, male predominance in NAFLD prevalence over females is believed to be influenced by the protective effects of estrogen in premenopausal women (2, 5). Several studies have underscored estrogen’s significant roles in antioxidative, anti-inflammatory, anti-apoptotic, and potential anti-fibrotic processes (5, 35–37). However, there have been no definitive reports on the relationship between serum vitamin A levels and all-cause mortality rates among different sexes. Although the exact mechanisms of these results remain unclear, clinical health management should pay particular attention to serum vitamin A levels in older adults (aged 60–85 years) and male populations.

The potential mechanisms underlying the relationship between serum vitamin A levels and all-cause mortality rates in NAFLD patients remain unclear. Oxidative stress, characterized by an imbalance between the generation of reactive oxygen species (ROS) and the clearance capacity of antioxidant systems such as superoxide dismutase and catalase, is believed to play a crucial role (4). Vitamin A exerts significant antioxidant effects in liver diseases and plays a critical role in controlling cell growth and differentiation (4, 16). It can inhibit the production of pro-inflammatory cytokines in macrophages, reduce inflammatory responses, suppress hepatocyte transformation, and inhibit liver cancer cell proliferation (38–41). Our findings showed that advanced liver fibrosis was most prevalent among patients with low serum vitamin A levels. Similarly, Song J et al. found that individuals with CLD who had the lowest retinol levels were significantly more likely to develop fibrosis and liver-related mortality compared to those with higher levels (31). A possible explanation for this is that the depletion of vitamin A may lead to oxidative stress-mediated damage observed in advanced liver disease. After liver injury, HSCs become activated and transform from vitamin A-rich, quiescent cells into proliferative and fibrogenic myofibroblasts. These activated cells produce excessive extracellular matrix, leading to liver fibrosis. Concurrently, there is a loss of characteristic perinuclear lipid droplets containing retinol (vitamin A), possibly leading to a loss of the ability of HSCs to store vitamin A (7, 11). However, our study revealed a U-shaped relationship between serum vitamin A levels and all-cause mortality in NAFLD patients. This may be due to excessive vitamin A metabolism, which could lead to an over-release of retinol-binding protein (RBP)/retinol complexes, thereby increasing lipid accumulation in liver cells and contributing to NAFLD progression. Moreover, excessive antioxidants might inhibit the induction of antioxidant defenses and the necessary pro-oxidative signals for tissue adaptation (16), potentially explaining why higher serum vitamin A levels are linked to increased all-cause mortality in NAFLD patients. Therefore, determining the most appropriate serum vitamin A levels is crucial.

Nevertheless, this study is subject to several limitations. Firstly, all measurements were conducted at baseline, and participants’ lifestyles and dietary habits may have changed during the long-term follow-up period, potentially affecting unmeasured variables that could influence the study outcomes. Secondly, we utilized the US FLI to assess hepatic steatosis and the FIB-4 score to evaluate hepatic fibrosis, which is not considered the gold standard for diagnosing NAFLD. Furthermore, despite adjusting for relevant covariates that could influence all-cause mortality rates, we cannot exclude the possibility of residual or unmeasured confounding factors affecting the study results. Lastly, there is a possibility that recall bias influenced self-reported data.

## 5 Conclusion

This study systematically investigated the association between serum vitamin A levels and all-cause mortality among NAFLD patients for the first time. The findings reveal a U-shaped correlation between serum vitamin A concentration and the risk of all-cause mortality among NAFLD patients in the United States. This finding offers new insights into the health management of patients with NAFLD, indicating that monitoring serum vitamin A levels may be important in clinical practice, particularly for men and older adults aged 60 and above, to reduce the risk of all-cause mortality.

## Data Availability

Publicly available datasets were analyzed in this study. This data can be found at: https://www.cdc.gov/nchs/nhanes/index.htm.

## Footnotes

^1^https://www.cdc.gov/nchs/nhanes/index.htm

## Glossary

NHANES: National Health and Nutrition Examination Survey
NCHS: National Center for Health Statistics
NAFLD: Non-alcoholic fatty liver disease
CDC: Centers for Disease Control and Prevention
NDI: National Death Index
CLD: Chronic liver disease
NASH: Non-Alcoholic Steatohepatitis
RCS: Restricted cubic spline
ROS: Reactive oxygen species
FIB4 score: Fibrosis-4 score
PNPLA3: Patatin-like Phospholipase Domain Containing 3
HbA1c: Glycated hemoglobin A1c
CRP: C-reactive protein
AST: Aspartate aminotransferase
ALT: Alanine aminotransferase
GGT: Gamma-glutamyl transferase
HDL: High-density lipoprotein
LDL: Low-density lipoprotein
ALP: Alkaline phosphatase
BMI: Body mass index
MET: Metabolic equivalent
FPG: Fasting blood glucose
TG: Triglycerides
TC: Total cholesterol
LDL-C: Low-density lipoprotein cholesterol
HDL-C: High-density lipoprotein cholesterol
HR: Hazard ratio
SE: Standard Error
OR: Odds ratio
